# Non–line-of-sight imaging over 1.43 km

**DOI:** 10.1073/pnas.2024468118

**Published:** 2021-03-03

**Authors:** Cheng Wu, Jianjiang Liu, Xin Huang, Zheng-Ping Li, Chao Yu, Jun-Tian Ye, Jun Zhang, Qiang Zhang, Xiankang Dou, Vivek K. Goyal, Feihu Xu, Jian-Wei Pan

**Affiliations:** ^a^Hefei National Laboratory for Physical Sciences at Microscale, University of Science and Technology of China, Hefei 230026, China;; ^b^Department of Modern Physics, University of Science and Technology of China, Hefei 230026, China;; ^c^Shanghai Branch, CAS Center for Excellence in Quantum Information and Quantum Physics, University of Science and Technology of China, Shanghai 201315, China;; ^d^Shanghai Research Center for Quantum Sciences, Shanghai 201315, China;; ^e^School of Earth and Space Science, University of Science and Technology of China, Hefei 230026, China;; ^f^School of Electronic Information, Wuhan University, Wuhan 430072, China;; ^g^Department of Electrical and Computer Engineering, Boston University, Boston, MA 02215

**Keywords:** non–line-of-sight imaging, optical imaging, computational imaging, computer vision

## Abstract

Non–line-of-sight (NLOS) imaging has the ability to reconstruct hidden objects from indirect light paths that scatter multiple times in the surrounding environment, which is of considerable interest in a wide range of applications. Whereas conventional imaging involves direct line-of-sight light transport to recover the visible objects, NLOS imaging aims to reconstruct the hidden objects from the indirect light paths that scatter multiple times, typically using the information encoded in the time-of-flight of scattered photons. Despite recent advances, NLOS imaging has remained at short-range realizations, limited by the heavy loss and the spatial mixing due to the multiple diffuse reflections. Here, both experimental and conceptual innovations yield hardware and software solutions to increase the standoff distance of NLOS imaging from meter to kilometer range, which is about three orders of magnitude longer than previous experiments. In hardware, we develop a high-efficiency, low-noise NLOS imaging system at near-infrared wavelength based on a dual-telescope confocal optical design. In software, we adopt a convex optimizer, equipped with a tailored spatial–temporal kernel expressed using three-dimensional matrix, to mitigate the effect of the spatial–temporal broadening over long standoffs. Together, these enable our demonstration of NLOS imaging and real-time tracking of hidden objects over a distance of 1.43 km. The results will open venues for the development of NLOS imaging techniques and relevant applications to real-world conditions.

## Significance

Non–line-of-sight (NLOS) imaging can recover details of a hidden scene from the indirect light that has scattered multiple times. Despite recent advances, NLOS imaging has remained at short-range verifications. Here, both experimental and conceptual innovations yield hardware and software solutions to increase NLOS imaging from meter to kilometer range. This range is about three orders of magnitude longer than previous experiments. The results will open avenues for the development of NLOS imaging techniques and relevant applications to real-world conditions.

In recent years, the problem of imaging scenes that are hidden from the camera’s direct line of sight (LOS), referred to as non–line-of-sight (NLOS) imaging (1–3), has attracted growing interest (4–14). NLOS imaging has the potential to be transformative in important and diverse applications such as medicine, robotics, manufacturing, and scientific imaging. In contrast to conventional LOS imaging (15–17), the diffuse nature of light reflected from typical surfaces in NLOS imaging leads to mixing of spatial information in the collected light, seemingly precluding useful scene reconstruction. To address these issues, optical techniques for NLOS imaging that have been demonstrated include transient imaging (4–8, 10, 11, 18), speckle correlations (9, 19), acoustic echoes (20, 21), intensity imaging (14), confocal imaging (22), occlusion-based imaging (23–27), wave-propagation transformation (28, 29), Fermat paths (30), and so forth (1–3). With few exceptions (14, 23–27), most of the techniques that reconstruct NLOS scenes rely on high-resolution time-resolved detectors and use the information encoded in the time-of-flight (TOF) of photons that scatter multiple times. Using such techniques, NLOS tracking of the positions of moving objects has been also demonstrated (12, 13). Despite this remarkable progress, NLOS imaging has been limited to short-range implementations, where the light path of the direct LOS is typically in the range of a few meters (1–3). Our interest is to demonstrate NLOS imaging over long ranges (i.e., kilometers), thus pushing its applications to real-life scenarios such as transportation, defense, and public safety and security.

The major obstacles to extending NLOS imaging to long ranges are signal strength, background noise, and optical divergence. Due to the three-bounce reflections and the long standoffs, the attenuation in long-range NLOS imaging is huge. Also, the weak back-reflected signal is mixed with ambient light, leading to poor signal-to-noise ratio (SNR). The sunlight contributes ambient noise, and the backscattering from the near-field atmospheric will also introduce high noise. Moreover, unlike long-range LOS imaging (31, 32), the signal detected at each raster-scanning point in NLOS imaging contains light reflected by all of the parts of the hidden scene. Consequently, it is more difficult to tolerate low SNR in NLOS imaging than in LOS imaging. On the other hand, the optical divergence over long range introduces a strong temporal broadening of the received optical pulses. Such broadening renders the idealizations of virtual sources and virtual detectors in previous short-standoff NLOS imaging experiments (19, 22, 28–30) inapplicable. Furthermore, previous NLOS experiments typically required high-precision timing measurements at picosecond scale—as obtained from a streak camera (4–6) or a single-photon avalanche diode (SPAD) detector (8, 10–13, 22, 28–30)—to recover hidden scenes. However, the temporal broadening in long-range situations can contribute time jitters on the order of nanoseconds (*SI Appendix*, Fig. S4). Lastly, long-range NLOS imaging needs a higher scanning accuracy, which will effect the resolution of the reconstruction results. All of these issues prevent the useful reconstructions of NLOS imaging over long standoffs.

We develop both hardware and software solutions to realize long-range NLOS imaging. First, we construct an NLOS imaging system operating at the near-infrared wavelength, which has the advantages (as compared to visible light) of low atmospheric loss, low solar background, eye-safety (33), and invisibility (*SI Appendix*, Fig. S2). Second, operating at near-infrared requires previously undescribed detection techniques, since the conventional Si SPAD does not work. To do this, we develop a fully integrated InGaAs/InP negative-feedback SPAD, which is specially designed for accurate light-detection and ranging applications (34). Third, we develop a high-efficiency optical receiver by employing a telescope with high coating efficiency and a single-photon detector with large photosensitive surface. The collection efficiency is >4.5 times higher than previous experiments (*SI Appendix*, Table S1). Fourth, we adopt a dual-telescope optical design for the confocal system (22) to reduce the backscattering noise, thus enhancing the SNR. The dual-telescope design can separate the illumination from detection and remove the use of beam splitter, thus allowing high illumination power and the optimization of receiver optics for high collection efficiency. Fifth, we optimize the system design to balance the collecting efficiency and the temporal resolution to realize high-resolution imaging (*SI Appendix*). We achieve a fine precision scanning with 46× and 28× magnification, where the scanning accuracy is as low as 9 microrads. Finally, we derive a forward model and a tailored deconvolution algorithm that includes the effects of temporal and spatial broadening in long-range conditions. With these efforts, we demonstrate NLOS imaging and tracking over a range up to 1.43 km at centimeter resolution. The achieved range is about three orders of magnitude longer than previous experiments (1, 2, 4–14, 18, 19, 22–30) (*SI Appendix*, Table S1).

## NLOS Imaging Setup

**Fig. 1.**
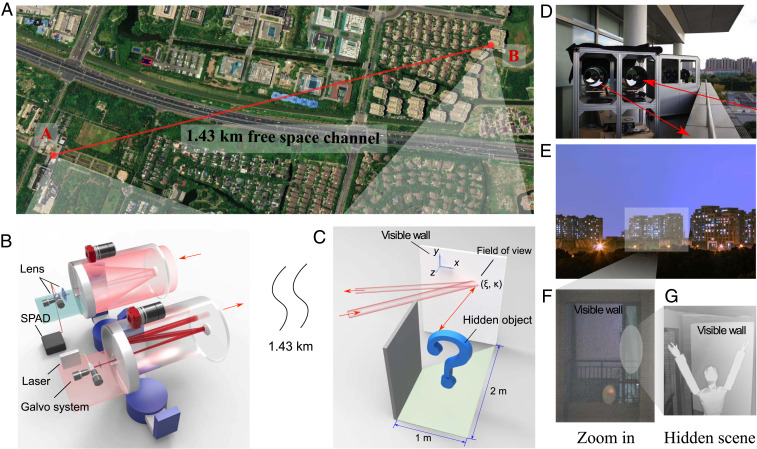
Long-range NLOS imaging experiment. (*A*) An aerial schematic of the NLOS imaging experiment over a (standoff distance of) 1.43-km free-space link, in which the setup is placed at A and the hidden scene is placed at B. (The geographic image is from Google Earth ©2020 Google.) (*B*) The optical setup of the NLOS imaging system, which consists of two synchronized telescopes for transmitter and receiver. A laser followed by galvo system and lenses are used for transmitting light pulses, while an InGaAs/InP SPAD together with a galvo system is used for collecting and detecting photons and recording their TOF information. (*C*) Schematic of the hidden scene in a room with a dimension size of 2 m×1 m. (*D*) An actual photograph of the NLOS imaging setup. (*E* and *F*) Zoomed-out and zoomed-in photographs of the hidden scene taken at location A, where only the visible wall can be seen. (*G*) Photograph of the hidden object, taken at the room located at B.

We implement NLOS imaging in the field, over an urban environment in the city of Shanghai. Fig. 1*A* shows an aerial view of the topology of the experiment. The imaging system is placed at location A ($31{}^{\circ}7^{\prime }33^{\prime \prime }$ N, $121{}^{\circ}32^{\prime }30^{\prime \prime }$ E), facing the hidden scene located at B ($31{}^{\circ}8^{\prime }6^{\prime \prime }$ N, $121{}^{\circ}31^{\prime }53^{\prime \prime }$ E) over a 1.43-km (determined by laser ranging measurement) free-space link. Note that this standoff distance is close to the maximal achievable range of our optical system (*SI Appendix*, Fig. S13). The hidden scene is an apartment on the 11th floor in a residential area with a size of about 2 m×1 m (Fig. 1*E*), where the objects are hidden from the field-of-view (FOV) of the NLOS imaging system, except for the visible wall (Fig. 1*F*).

The optical NLOS imaging setup, shown in Fig. 1*B*, utilizes commercial off-the-shelf devices that operate at room temperature, where the source is a standard fiber laser, and the detector is a compact InGaAs/InP single-photon avalanche diode (SPAD). This shows its potential for real-life applications. In particular, the whole system is operated at 1,550.2 nm, and it adopts a dual-telescope confocal optical design with two identical Schmidt–Cassegrain optical telescopes, each of which has a diameter of D=280 mm and a focal length of f=2.8 m. In the transmitter, pulsed light is generated by a fiber laser (BKtel HFL-300am) at the pulse width of ∼500 ps, 300-mW average power, and 1-MHz repetition frequency. The pulses pass through an 11-mm collimator, a two-axis raster-scanning galvo mirror, and an optical lens with 30-mm focal length. Then, the pulses go into the transmitting telescope and are sent out over the free-space link to the visible wall at location B. For the optical receiver, diffuse photons are collected by the receiving telescope and guided to a lens followed by the receiving galvo mirror. Then, the photons are coupled to a multimode fiber and directed to a homemade, compact, multimode fiber-coupled, free-running InGaAs/InP SPAD detector (34), which has ∼20% system detection efficiency, ∼210-ps timing jitter, 2.8 K dark count rate. The photon-detection signals from the SPAD are recorded and time tagged by a time-correlated single photon counting module with a time resolution of 32 ps.

To achieve a high-efficiency, low-noise NLOS system, we implement several optimized optical designs. In the receiving system, we utilize conversion lenses with focal lengths of 60 and 100 mm, and we magnify the equivalent aperture angle of the telescope, which matches with the multimode fiber with a core diameter of 62.5 μm and NA = 0.22 (*SI Appendix*). Such a design not only achieves a high coupling efficiency but also reduces the background noise from stray light. The total system efficiency, including transmission efficiency of lenses and galvo mirrors (40%), fiber coupling efficiency (50%), and SPAD detector efficiency (20%), is ∼4%. Meanwhile, the 62.5-μm fiber ensures a proper FOV of 14 cm (at 1.43-km relay wall) per pixel for high-resolution NLOS imaging. Moreover, the galvo system is placed between the two lenses so that the rotation angle of the galvo can be well determined by the lens and the telescope.

In long-range conditions, a standard coaxial design of the confocal NLOS imaging system (22) will present high background noise due to the direct backscatter. When the illumination light passes through the system’s internal mirrors and telescope, it will produce strong local backscattered noise due to their limited coating. This noise is much higher than the NLOS signal photons that travel back after three bounces. Besides, the near-field atmosphere will also contribute direct backscatter. To resolve these issues, we designed a dual-telescope optical system, where the illuminator and the receiver utilize two different telescopes to remove the cross-talk between them. The dual-telescope system can also avoid the beam splitter (22), which allows the use of high illumination power and optimization of the receiver optics for high collection efficiency. The two telescopes are carefully matched and automatically synchronized for each raster-scanning position. Furthermore, the central positions of each illuminating point and receiving FOV, projected on the visible wall, are set to be slightly separated in order to reduce the effect of first-bounce light. This separation is controlled at ∼9.8 cm, which is much smaller than the distance between the hidden object and the visible wall (∼2 m); so the spherical geometry resulting from coaxial alignment is an accurate approximation.

For the choices of the FOVs of the transmitting and receiving systems, there is a tradeoff between the collection efficiency and the system timing jitter. In our experiment, we perform an optimization to choose both FOVs at a diameter of ∼14 cm projected on the visible wall, where the receiving FOV matches the numerical aperture and the core diameter of the multimode fiber (*SI Appendix*). The transmitting and receiving telescopes raster-scan a 64×64 grid with a total area of 0.85 m × 0.85 m on the visible wall. The distance between the central positions of two neighboring scan points is set to be ∼1.3 cm. This is achieved by designing the two optical telescope systems to magnify the rotation angle of the two galvo systems.

## Forward Model and Reconstruction Algorithm

Inspired by confocal NLOS imaging based on the light-cone transform (LCT) (22), we derive an image formation algorithm that is tailored for long-range outdoor conditions. The forward model takes two additional parameters into consideration: 1) the system’s time jitter $\sigma_{t}$, which includes time-broadening contributions (35) from the laser pulse width, the detector’s jitter, the optical divergence, etc.; and 2) the standard deviation (SD) of the receiver’s size of FOV projected onto the visible wall $\sigma_{x}\times \sigma_{y}$. Similar to most NLOS imaging methods, our model makes the following assumptions: the visible wall and the hidden object are both Lambertian and the influence of the angle of the Lambertian scattering can be ignored, and the photons that bounce more than three times can be ignored.

### Forward Model.

A topology of the imaging formation is shown in *SI Appendix*, Fig. S1. We consider a confocal NLOS imaging system where the receiver’s and transmitter’s FOVs overlap. We assume that the laser and the detector raster-scan an m×m grid at the same points. Let (ξ,κ) denote the scanning points on the visible wall and (x,y,z) denote the patch on the object with reflectivity α(x,y,z). The detection rate as a function of time for each grid position is a three-dimensional (3D) signal s(ξ,κ,t) modeled as

$$\begin{array}{cc} & s\left(\xi ,\kappa ,t\right)=g_{xy}{*}_{xy}g_{t}{*}_{t}\\ & ∭\frac{1}{r^{4}\left(x-\xi ,y-\kappa ,z\right)}\alpha \left(x,y,z\right)\;\delta \left(2r-ct\right)\;dx\;dy\;dz, \end{array}$$ [1]

where

$$\begin{array}{cc} r\left(x-\xi ,y-\kappa ,z\right) & =\sqrt{{\left(x-\xi \right)}^{2}+{\left(y-\kappa \right)}^{2}+z^{2}},\\ g_{t} & =\exp\;\left(-\frac{t^{2}}{2\sigma_{t}^{2}}\right),\\ g_{xy} & =\exp\;\left(-\frac{\xi^{2}}{2\sigma_{x}^{2}}-\frac{\kappa^{2}}{2\sigma_{y}^{2}}\right). \end{array}$$

Here, r denotes the distance between the scanning point (ξ,κ) on the visible wall and the point (x,y,z) on the hidden object; $g_{t}$ is the temporal distribution of the whole transceiver system, modeled by a Gaussian function with SD $\sigma_{t}$; $g_{xy}$ describes the spatial distribution of the receiver’s FOV projected on the visible wall, which can be modeled by a two-dimensional (2D) Gaussian distribution with the spatial size (SD) of $\sigma_{x}\times \sigma_{y}$; ${*}_{xy}$ represents convolution with respect to the spatial variables; and ${*}_{t}$ represents convolution with respect to the time variable. Note that several scalar factors are absorbed into α(x,y,z), such as the detection efficiency and normalization factors for $g_{t}$ and $g_{xy}$.

According to the properties of the Dirac delta function and the change of the variables $u=z^{2}$ and $v={\left(ct/2\right)}^{2}$ adopted in the LCT model (22), Eq. **1** can be rewritten as

$$\begin{array}{cc} & s\left(\xi ,\kappa ,2\sqrt{v}/c\right)\;=\\ & \underset{G\left(\xi ,\kappa ,v\right)}{\underset{︸}{\exp\;\left(-\frac{\xi^{2}}{2\sigma_{x}^{2}}-\frac{\kappa^{2}}{2\sigma_{y}^{2}}\right){*}_{xy}\exp\;\left(-\frac{2v}{c^{2}\sigma_{t}^{2}}\right)}}{*}_{v}\tilde{s}\left(\xi ,\kappa ,v\right), \end{array}$$ [2]

where

$$\begin{array}{cc} & \tilde{s}\left(\xi ,\kappa ,v\right)\;=\\ & v^{-3/2}∭\underset{\tilde{\alpha }\left(x,y,u\right)}{\underset{︸}{\frac{\alpha \left(x,y,\sqrt{u}\right)}{2\sqrt{u}}}}\underset{H\left(\;\xi -x,\kappa -y,v-u\right)}{\underset{︸}{\delta \left({\left(\xi -x\right)}^{2}+{\left(\;\kappa -y\right)}^{2}+u-v\right)}}dx\;dy\;du. \end{array}$$

Upon discretizing time (raster-scan points are already discrete), the pair of convolutions in Eq. **2** has a discrete representation, s=G(ξ,κ,v)*H(α;ξ,κ,v). Here, G is a 3D spatiotemporal matrix representing the facula intensity distribution on the visible wall and the system jitter distribution in temporal domain; H models the light-cone transform in confocal NLOS imaging. From the theory of photodetection, the photons detected by the SPAD follow an inhomogeneous Poisson distribution. Therefore, the image formulation of this long-range confocal NLOS imaging is

Y∼Poisson(Aα+B), [3]

where $Y\in R^{n_{x}n_{y}n_{t}}$ represents the discretized measured photon count detected by scanning point $\left(n_{x},n_{y}\right)$, $n_{t}$ discrete histogram time bin. $A\in R^{n_{x}n_{y}n_{t}\times n_{x}n_{y}n_{z}}$ encodes the discretized version of the imaging formulation in Eq. **1**. $\alpha \in R^{n_{x}n_{y}n_{z}}$ is the distribution of the hidden object’s reflectivity. Furthermore, $B\in R^{n_{x}n_{y}n_{t}}$ represents the dark count of the detector and the background noise.

### Reconstruction.

The goal of imaging reconstruction is to estimate α, which contains both intensity and depth information, from the measurement data S. To reconstruct α, we describe the inverse problem as a convex optimization. Let L(α;S,H,G,B) denote the negative log-likelihood data-fidelity function from Eq. **3**. The computation of the regularized maximum-likelihood estimate can be written as the convex optimization problem

$$\begin{array}{cc} \underset{\alpha }{\mathrm{minimize}} & \;\Phi \left(\alpha \right)=L\left(\alpha ;S,H,G,B\right)+\lambda \|{\alpha \|}_{\text{TV}},\\ \text{subject to} & \alpha_{i,j,k}\geq 0,\forall i,j,k. \end{array}$$ [4]

Here, the constraint $\alpha_{i,j,k}\geq 0$ comes from the nonnegativity of the reflectivity, and the total variation (TV) is exploited as the regularizer, where λ is the regularization parameter.

We develop an iterative 3D deconvolution algorithm, modified from Sparse Poisson Intensity Reconstruction Algorithms–Theory and Practice (SPIRAL-TAP) (36), to obtain an approximate solution of the inverse problem in Eq. **4**. This NLOS problem is different from the standard LOS algorithms (37, 38). Our reconstruction algorithm—SPIRAL3D—employs sequential quadratic approximations to the log-likelihood objective function Φ(α). SPIRAL3D extends the conventional SPIRAL-TAP from a 2D matrix to a 3D matrix. That is, it performs the iterative optimization directly for 3D matrices that include both the spatial and temporal information, in order to match the convolutional operators in the forward model of Eqs. **1**, **2**, and **3** (39). The convergence of this iteration method has been proved in ref. 36.

## NLOS Imaging Results

In the long-range case shown in Fig. 1, the temporal resolution of received photons after three bounces is characterized to be a full width at half-maximum of 1.1 ns in good weather conditions (*SI Appendix*, Fig. S4). In the experiment, we observe that the total attenuation for the third bouncing is ∼160 dB over the 1.43-km link, when a typical hidden object (∼0.6 m × 0.4 m size) is placed about 1 m away from the visible wall. On average, for each emitted pulse, the received mean photon numbers for the first and third bounce are about 0.048 and 0.0003. During data collection, for each raster-scanning point, we normally operate the system with an exposure time of 2 s, where we send out laser pulses with a total number of $∼4.6\times 10^{18}$ photons and collect ∼674 returned third-bounce photon counts. The SNR for the third bouncing is about 2.0. The data are mainly collected at night, because of relatively stable temperature and humidity (*SI Appendix*, Fig. S3). We also perform measurements in daylight. By using proper spectral filters, we observe similar SNRs (*SI Appendix*, Fig. S12). For each scanning position, a photon-counting histogram mainly consists of two peaks in the temporal domain (see a typical sequence in *SI Appendix*, Fig. S5). The first peak records the photons reflected by the visible wall (first bounce), and the second peak records the photons through three bounces (third bounce). The time difference between first-bounce and third-bounce photons is extracted as the signal for NLOS image reconstructions.

**Fig. 2.**
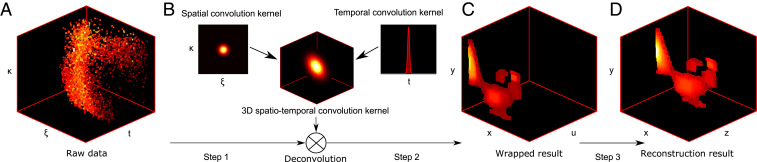
The reconstruction procedure of our approach. (*A*) The raw-data measurements are resampled along the time axis. (*B*) The spatiotemporal kernel is applied to the 3D raw data to solve the deconvolution with the SPIRAL3D solver. (*C*) Transforming the coordinate along depth from u to z to recover the hidden scene. (*D*) The final reconstructed result.

Fig. 2 illustrates the process of the reconstruction for the hidden mannequin. The process generally involves three steps: 1) setting the raw data as the initial value of the iteration; 2) applying the spatiotemporal kernel to solve the 3D deconvolution with the SPIRAL3D solver; and 3) transforming the coordinate along depth from u to z to recover α. With these three steps, the shape and albedo of the hidden mannequin can be reconstructed successfully.

**Fig. 3.**
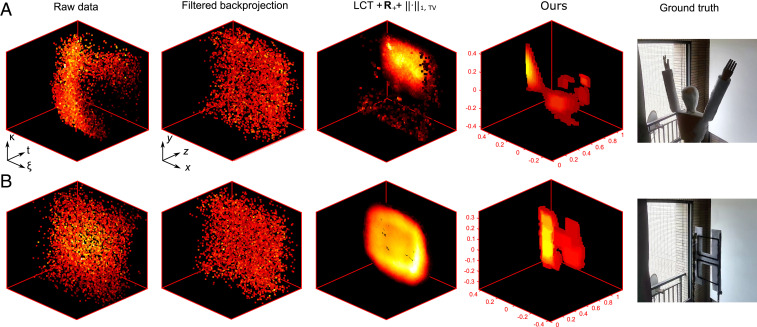
Comparison of the reconstructed results with different approaches. The NLOS measurements are computed by filtered back-projection with a 3D Laplacian of Gaussian filter (40), LCT with an iterative ADMM-based solver (22), and our proposed algorithm. (*A*) The reconstructed results for the hidden scene of mannequin. (*B*) The reconstructed results for the hidden scene of letter H.

Fig. 3 shows the results for two distinct hidden objects, which are computed with different types of NLOS reconstruction approaches, including filtered back-projection with a 3D Laplacian of Gaussian filter (40), LCT with an iterative solver based on Alternating Direction Method of Multipliers (ADMM) (22), and the algorithm proposed herein. The proposed algorithm recovers the fine features of the hidden objects, allowing the hidden scenes to be accurately identified. The other approaches, however, fail in this regard. These results clearly demonstrate that the proposed algorithm has superior performance than state-of-the-art approaches for NLOS reconstruction of hidden targets in outdoor and long-range conditions. In the experiment, we can resolve two hidden objects of ∼9.4 cm apart, which behaves better than the approximate resolution bound (*SI Appendix*). *SI Appendix*, Figs. S6 and S10 show additional results such as 3D scenes that contain multiple objects with different depths. For a longer distance, the optical divergence introduces larger spatial–temporal broadening, which will deteriorate the accuracy of the reconstructed image (*SI Appendix*, Fig. S14).

## NLOS Tracking Results

**Fig. 4.**
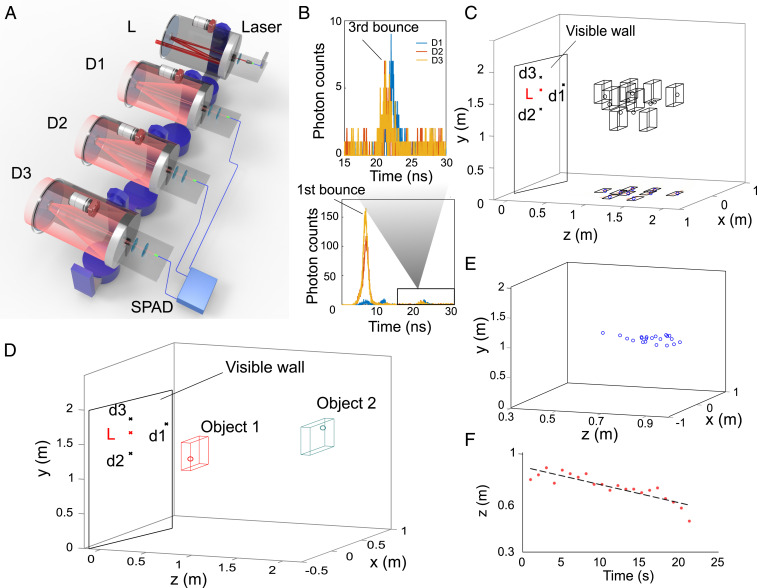
NLOS real-time detecting and tracking experiment over 1.43 km. (*A*) The setup includes one telescope and a pulsed laser for transmitting light (L) and three telescopes and SPADs for detecting light (D1, D2, D3). (*B*) A representative raw measurement contains the first- and third-bounce photon counts. (*C*) Detection of the various positions for a single hidden object. (*D*) Detection of the positions for two simultaneous hidden objects. (*E*) The real-time tracking for a hidden moving object at a frame rate of 1 Hz. (*F*) The real-time tracking speed for a hidden moving object, where the retrieved speed is 1.28 cm/s, which matches the actual speed of 1.25 cm/s.

We perform experiments to test NLOS tracking for the positions of hidden objects in real time over 1.43 km. Obtaining the position of hidden objects is sufficient for a number of applications, and earlier remarkable experiments have proved the feasibility for NLOS tracking up to 50 m (12, 13). As shown in Fig. 4*A*, our experiment is realized with three receiver telescopes simultaneously observing three distinct positions D1, D2, and D3, while a transmitter telescope transmits laser pulses at position L on the visible wall. These four positions on the visible wall are optimized to achieve high-resolution detecting of the hidden objects (Fig. 4 *C* and *D*). The collected photons from the three receiver telescopes are coupled to multimode fibers and detection systems, which include three-channel InGaAs/InP SPADs and TDCs. By detecting the TOF information of the photons reflected by the hidden object, one can retrieve both the coordinates and the speed of the target. Following refs. 12 and 13, we use the Gaussian model to correct the time jitter and adopt the estimation method of the maximum likelihood for ellipsoid curves.

The results of NLOS detection and tracking are shown in Fig. 4. We implement the detection of a single hidden object for difference positions (Fig. 4*C*) and the detection of two hidden objects (Fig. 4*D*). In the experiment, we show the ability to retrieve hidden objects at different hidden distances to the visible wall. The agreement between the joint probability density function and the object’s true position shows that our system is able to locate a stationary person up to ∼1.8 m. We perform 10 measurements for each position of the hidden object and observe that the SD is ∼ [10, 10, 2.5] cm and the error (or accuracy) between the mean value and the actual position is ∼ [7, 8, 3] cm, at the hidden distance of 1 m. These results are comparable to previous experiments (12, 13). Furthermore, we demonstrate real-time tracking (Fig. 4 *E* and *F*) of a moving object at a frame rate of 1 Hz. Fig. 4*E* shows the retrieved positions of the moving object at different times. The retrieved speed is 1.28 cm/s according to the fitting result in Fig. 4*F*, which matches the actual speed of the hidden object of 1.25 cm/s. There results demonstrate the potential for NLOS detection and tracking in real time over a long range.

## Discussion

In summary, we demonstrate in this work NLOS imaging and tracking of a hidden scene at long ranges up to 1.43 km. The computed images allow for NLOS target recognition and identification at low light levels and at the invisible wavelength with eye safety. (It is noted that our aim is to extend the standoff distance of NLOS techniques while the scale of the hidden scene is similar to the previous work at room scale.) In the future, advanced devices such as femtosecond lasers, high-efficiency superconducting detectors (41), and SPAD array detectors (42) can be used to boost the imaging resolution, increase the imaging speed, and extend the scale of the hidden scene. The atmospheric turbulence will be another consideration when going toward longer ranges. Overall, the proposed high-efficiency confocal NLOS imaging/tracking system, noise-suppression method, and long range-tailored reconstruction algorithm open opportunities for long-range, low-power NLOS imaging, which is important to move NLOS imaging techniques from short-range validations to outdoor and real application.

## Materials and Methods

### Supporting Details.

In *SI Appendix*, we provide more details on both hardware and software. For hardware, we mainly discuss the optimized system design and some calibrations; for software, we include a more detailed forward model and some simulation results. Full information regarding materials and methods is available in *SI Appendix*.

## Supplementary Material

Supplementary File

Supplementary File

## Data Availability

All data and processing code supporting the findings of this study have been deposited in GitHub (https://github.com/quantum-inspired-lidar/NLOS_imaging_over_1.43km).
