# Supplementary material for: Non–line-of-sight imaging over 1.43 km
Source: Proc Natl Acad Sci U S A. 2021 Mar 3;118(10):e2024468118. doi: 10.1073/pnas.2024468118 (PMC7958383; doi:10.1073/pnas.2024468118)
Supplement: Supplementary File [file pnas.2024468118.sapp.pdf]

Supplementary Information for

Non-line-of-sight imaging over 1.43 kilometers

Cheng Wu, Jianjiang Liu, Xin Huang, Zheng-Ping Li, Chao Yu, Jun-Tian Ye, Jun Zhang, Qiang Zhang, Xiankang Dou, Vivek K. Goyal, Feihu Xu and Jian-Wei Pan

Feihu Xu and Jian-Wei Pan.
E-mail: feihuxu@ustc.edu.cn and pan@ustc.edu.cn.

This PDF file includes:

Supplementary text
Figs. S1 to S14
Table S1
Legend for Movie S1
SI References

Other supplementary materials for this manuscript include the following:

Movie S1

Supporting Information Text

A. Optimized system design. For NLOS imaging, the echo light signal, scattered from the hidden scene, decreases rapidly with the direct distance R from the imager to the visible wall and the hidden distance r from the visible wall to the hidden object. Here we consider a confocal NLOS imaging system where the receiving field of view (FOV) and the transmitting FOV overlap. For an imaging system with laser power P_t and telescope aperture D, the received signal can be written as(1)

$$P_r = P_t \alpha_v^2 \alpha \frac{D^2 A_{obj} A_{\text{FOV}}}{8\pi^3 r^4 R^2} \cdot \eta_{sys} \cdot (1 - \varepsilon_b(R)) \cdot \exp\left(-2\int_0^R \varepsilon_e(r)dr\right), \quad [1]$$

using visible wall reflectivity α_v, hidden object reflectivity α, scattering area A_{obj}, FOV area A_{FOV}, and receiver system efficiency η_{sys}. Here, we also consider the atmospheric effect: ε_b is the volume backscattering coefficient, ε_e is the atmospheric extinction coefficient. From Eq. (1), the collected photon number, thus SNR, is *quadratically proportional* to FOV.

As in shown in Fig. S7, the spatial broadening of FOV, σ_{FOV}, leads to the broadening in temporal response, which can be calculated as

$$\frac{c \cdot \sigma_t'}{2} \simeq \sigma_{\text{FOV}} \cdot \cos\psi, \quad [2]$$

where $\psi \in (0, \frac{\pi}{2}]$ is the angle between the light path (reflected by the hidden object) and the visible wall. Therefore, by ignoring the effect of weather and air turbulence, the *total* time jitter (or temporal response), γ_{total} (FWHM), can be written as

$$\gamma_{total} = 2\sqrt{2\ln 2}\sqrt{(2\sigma_{\text{FOV}}\cos\psi/c)^2 + \sigma_t^2}. \quad [3]$$

According to the resolution limits derived in ref.(2), the resolution of NLOS reconstruction is *inversely proportional* to the temporal response.

Clearly, the selection of FOV has a trade-off between SNR and imaging resolution. By numerical simulation and experimental measurement, we carefully vary the sizes of FOV and perform the corresponding NLOS reconstructions. In experiment, we select FOV on the order of tens of centimeters (cm) in order to collect sufficient photon counts and realize a decent resolution in cm scale. For the choice of the telescope, a larger aperture will have a higher collection efficiency, which is desirable for long rang NLOS. However, the price will be much higher and a larger-size telescope also concerns the practicality. For demonstration purpose, we adopt a commercial off-the-shelf telescope with a diameter of $D = 280$ mm, which has been widely used in standard long range LiDAR experiments(3, 4). We use a multimode optical fiber with a core diameter of $d_f = 62.5$ μm and NA $= 0.22$ to collect echo photons. At the direct distance $R = 1.43$ km, the aperture angle is $\theta_a = D/R = 196$ μrad. According to the optical invariant, we obtain the relationship between FOV and the receiving angle θ_r of the fiber, FOV $= d_f \cdot \theta_r/\theta_a = 0.3189 \cdot \theta_r \leq 0.3189 \cdot (2 \cdot NA) = 14$ cm. We adjust the lens group to fully utilize the receiving angle of the fiber to realize a final FOV of 14 cm on the relay wall, which is characterized by the beam divergence angle. With this FOV, we achieve a NLOS resolution of ~9.4 cm with our algorithm (see below).

B. Calibration. We calibrated the total time jitter (or temporal response) of the NLOS system over the 1.43-km link by sending out the laser pulses to the visible wall and measuring the LOS back reflections. The results are illustrated in Fig. S5, which shows a total system jitter FWHM of γ_{total} ~1.1 ns. It mainly includes the SPAD detector γ_{det}=210 ps, the laser pulse γ_{pulse}=500 ps and the temporal broadening due to the 14-cm FOV γ_{FOV}=855 ps.

Two 2-axis scanning galvanometers were used in the transmitter system and the receiver system respectively to perform confocal raster-scanning. Both the field of regard (FoR) along x axis and y axis are set to ~594 μrad, which corresponds to a square area of about 85 cm × 85 cm (projected to the visible wall). Suppose we start scanning from the point located in the top left corner of the area, and consider the scanning along one of x and y axis. Let β be the rotating angle from the starting point to current point, where 0 μrad $\leq \beta \leq$ 594 μrad. And let d (unit: m) describe the transverse range between these two points. According to the geometric derivations, $d \approx 1430 \times \frac{\sin\beta}{\sin\phi}$, where ϕ is a rough angle between the laser beam and the x or y axis and $\beta \ll \phi$. Furthermore, since β is a small number, the relationship between d and β can be approximated by a linear function, i.e., we can perform the scanning evenly by linearly changing the scanning angle along each axis. Thus, once we set the coordinate of one fixed scanning point as the starting point, the coordinates of other points can be calculated easily.

According to American National Standards Institute, Z136.1-2000(5, 6), we can evaluate the eye safety condition. For our 1550 nm-based system, two rules for the pulsed laser can be taken into consideration. Rule1: Singe-pulse maximum permissible exposure (MPE). Rule 1 indicates that the output energy per pulse of the system cannot exceed 7.85×10^{-3}J. Rule 2: Average power MPE for thermal and photochemical hazards. Rule 2 indicates that the output energy per pulse of the system cannot exceed 1.57×10^{-9}J.

Obviously, Rule 2 gives a stricter bound. Now, we calculate the effective output energy per pulse of our system considering the beam diameter D_L and the limiting aperture diameter D_f (a constant depending on the structure of human eyes),

$$Q_f = Q_0[1 - e^{-(D_f/D_L)^2}]$$

Given that the average laser power is 300 mW and the repetition frequency is 1 MHz, the output energy per pulse $Q_0 = 0.3$ μJ. Then considering the D_L of 7 cm (from experimental calibration of the system) and the D_f of 1mm, $Q_f = 6 \times 10^{-11}$J. Therefore, $Q_f < rule2 < rule1$. Our system can be assessed as a relatively eye-safe system.

C. Forward Model. Based on ref.(2), without considering the spatial and temporal broadening, the signal that NLOS system received can be written as:

$$s(\xi,\kappa,t) = \iiint \frac{1}{r^4(x-\xi, y-\kappa, z)} \alpha(x,y,z)\, \delta(2r-ct)\, dx\, dy\, dz, \qquad [4]$$

where

$$r(x-\xi, y-\kappa, z) = \sqrt{(x-\xi)^2 + (y-\kappa)^2 + z^2}.$$

Here, $(\xi, \kappa, 0)$ is the coordinate of the scanning point, (x, y, z) is the coordinate of the hidden object, and r is the distance between the scanning point and the hidden object, t represents the traveling time of the light, c represents the speed of light.

In our new forward model, we include *both* temporal and spatial broadening into Eq. (4). Firstly, as shown in Figure S1, we consider the spatial broadening due to FOV, where the spatial divergence can be assumed to be a 2D Gaussian spot. Assuming the standard deviations of the 2D Gaussian function are σ_x and σ_y, the optical intensity of a certain point $(\xi', \kappa', 0)$ in the FOV can be written as,

$$g_{xy} = P \exp\left(-\frac{(\xi-\xi')^2}{2\sigma_x^2} - \frac{(\kappa-\kappa')^2}{2\sigma_y^2}\right). \qquad [5]$$

Here, P represents the central power of the spot, which depends on the system's parameters and can be recognized as a constant. For convenience, P will be ignored in later derivations. Hence, considering the spatial broadening, the total signal $s_{spot}(\xi, \kappa, 0)$ is the sum of the signal $s(\xi', \kappa', 0)$ from each point in the spot, i.e.,

$$s_{spot}(\xi,\kappa,0) = \int s(\xi',\kappa',0) \times \exp\left(-\frac{(\xi-\xi')^2}{2\sigma_x^2} - \frac{(\kappa-\kappa')^2}{2\sigma_y^2}\right) d\xi' d\kappa' \qquad [6]$$

Obviously, Eq. (6) can be written as a convolution between $s(\xi, \kappa)$ and $g_{xy}(\xi, \kappa)$,

$$s_{spot}(\xi,\kappa,0) = g_{xy} *_{xy} s$$

Here, $*_{xy}$ represents convolution with respect to the spatial coordinates in x and y.

Similarly, by considering the temporal broadening, the signal $s_{time}(\xi, \kappa, 0)$ can be written as a convolution between $s(\xi, \kappa, 0)$ and a Gaussian function g_t along time-axis,

$$s_{time}(\xi,\kappa,0) = s *_t g_t \qquad [7]$$

$$g_t = exp\left(-\frac{t^2}{2\sigma_t^2}\right)$$

Here, $*_t$ represents convolution with respect to the time coordinate. By having both temporal and spatial broadening effect, we arrive at the equation (1) in main text.

D. Simulations. We present numerical simulation results to evaluate the resolution of our algorithm (Fig. S8 and S9) and the algorithm's ability to reconstruct 3D targets (Fig. S10 and S11).

To evaluate the reconstructed resolution, we simulate the NLOS reconstruction of a resolution chart. The results are shown in Figs. S8 and S9. The diagram of this simulation experiment is shown in Fig. S1. The size of the hidden space is $1m \times 1m \times 1.2m$, the distance between the hidden scene and the visible wall is $\sim$0.7 m, and the size of the hidden resolution chart is 1 m $\times$ 1 m. This chart consists of 64 $\times$ 64 pixels, which means that each pixel's area is $\frac{1}{64}$ m $\times$ $\frac{1}{64}$ m. In the chart, the distances between the two adjacent horizontal stripes in the top-left part and bottom-right part are 3 pixels ($\frac{3}{64}$ m) and 4 pixels ($\frac{4}{64}$ m), respectively. The distances between the two adjacent vertical stripe in the top-right part and bottom-left part are 5 pixels ($\frac{5}{64}$ m) and 6 pixels ($\frac{6}{64}$ m), respectively.

Fig. S8 shows the reconstruction results for NLOS data with system time jitter ranging from 100 ps to 1000 ps. The spatial divergence is fixed at $\frac{1}{64}m$. It can be seen that the spatial resolution of the reconstructed results becomes worse with the increase of time jitter. At each time jitter, our method performs better than other approaches(2, 7). When the time jitter is 1000 ps, we can hardly see anything from other methods. In contrast, our approach has the ability to recover the large vertical stripes. In this case, the transversal resolution can reach $\sim$9.4 cm, whereas the resolution bound calculated using the method in ref.(2) is $\Delta x \sim$ 26 cm. Similarly, Fig. S9 shows the reconstruction for NLOS data with different spatial divergences, varying from $\frac{3}{64}$ m to $\frac{9}{64}$ m. The time jitter used in this simulation experiment is 100 ps. It is obvious that the quality of the result becomes worse with the increase of the spatial divergences. When the spatial divergence is $\frac{9}{64}$ m, the transversal resolution bound(2) is calculated to be $\Delta x = \frac{c\sqrt{w^2+z^2}}{2w}\gamma_{total} = \frac{c\sqrt{w^2+z^2}}{2w} \cdot 2\sqrt{2ln2}\sqrt{(\frac{2\sigma_{FOV}cos\psi}{c})^2 + \sigma_t^2} = 29$ cm (where $2\sigma_{FOV} = \frac{9}{64}\ m, cos\psi = 1$ and $\sigma_t = 100\ ps$). In contrast, our method reaches a resolution of $\sim$9.4 cm.

Overall, since our forward model and reconstruction algorithm have taken the spatial-temporal broadening priors into account, the reconstructed result can mitigate the influence of the broadening effect. This is the reason why we can get reconstruction results (in Fig. S8 and S9) better than the predicted resolution which considers the broadening effect. Whereas, our reconstruction can't beat the predicted resolution which does not include the broadening effect.

Fig. S10 shows the reconstruction result of a 3D scene with four letters U, S, T and C of different reflectivity and depth. The four letters have different reflectivity of 0.5, 1, 0.7 and 0.9, and the depths of these four letters are 1.1 m, 0.9 m, 0.7 m and 0.5 m, respectively. The size of these four letters are approximately $\frac{20}{64}$ m $\times$ $\frac{25}{64}$ m. The time jitter is set to be 100 ps and the spatial divergence is $\frac{3}{64}m$. Fig. S11 shows the reconstruction results of a mannequin, which is from the data of LCT(2). From these two simulations, the capability of our algorithm to reconstruct 3D scenes are demonstrated.

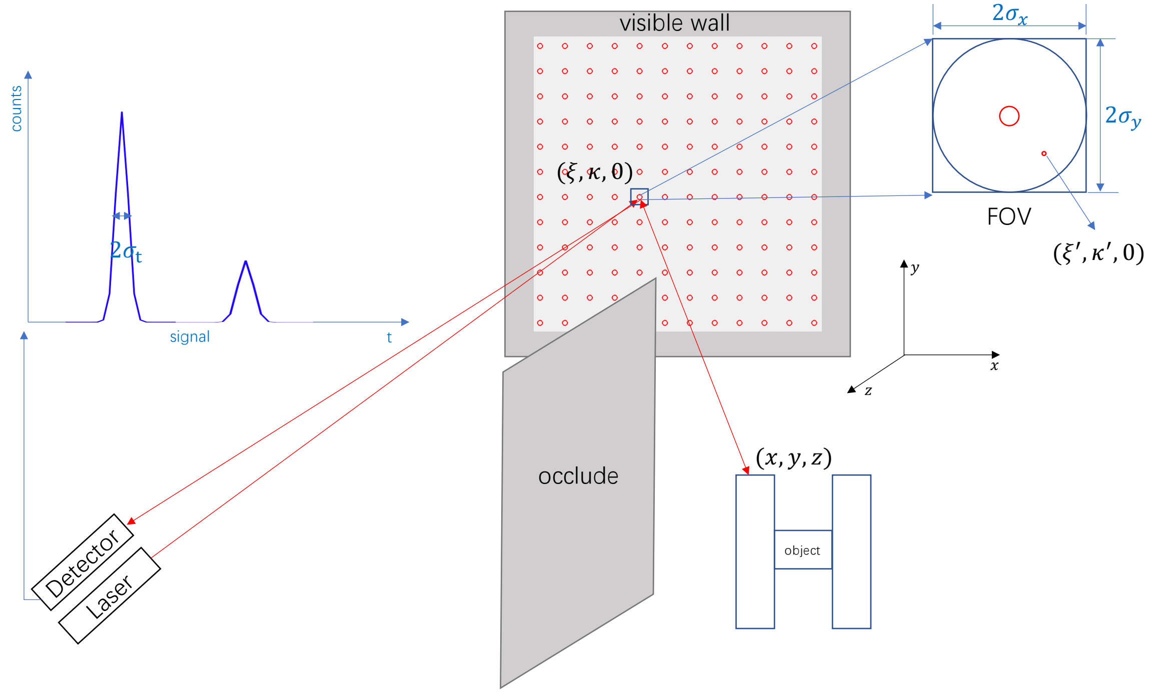

Fig. S1. Schematic diagram of the experiment. The laser and the detector confocal raster-scan an $m \times m$ grid on the visible wall. $(\xi, \kappa, 0)$ represents the scanning points on the visible wall and (x, y, z) represents the points on the object. The time jitter of the whole transceiver system is modelled by a Gaussian distribution with the standard deviation σ_t. Each scanning has a FOV whose spatial distribution modelled by a 2D Gaussian distribution with the spatial size of $2\sigma_x \times 2\sigma_y$.

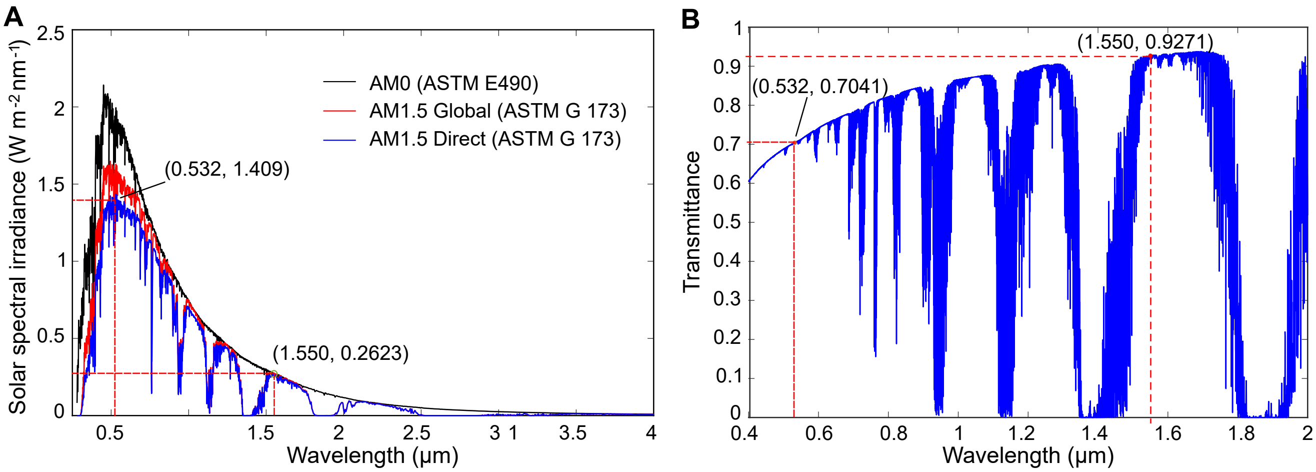

Fig. S2. (*A*) Solar spectral irradiance. The solar background irradiance is about 0.26 at 1.55 μm and 1.41 at 0.53 μm. (*B*) Spectral transmittance in a km-range horizontal atmosphere. The atmospheric transmittance is about 0.93 at 1.55 μm and 0.70 at 0.53 μm. Near-Infrared wavelength at 1.55 μm has advantages of *low* solar background noise and *high* transmittance as compared to visible wavelength.

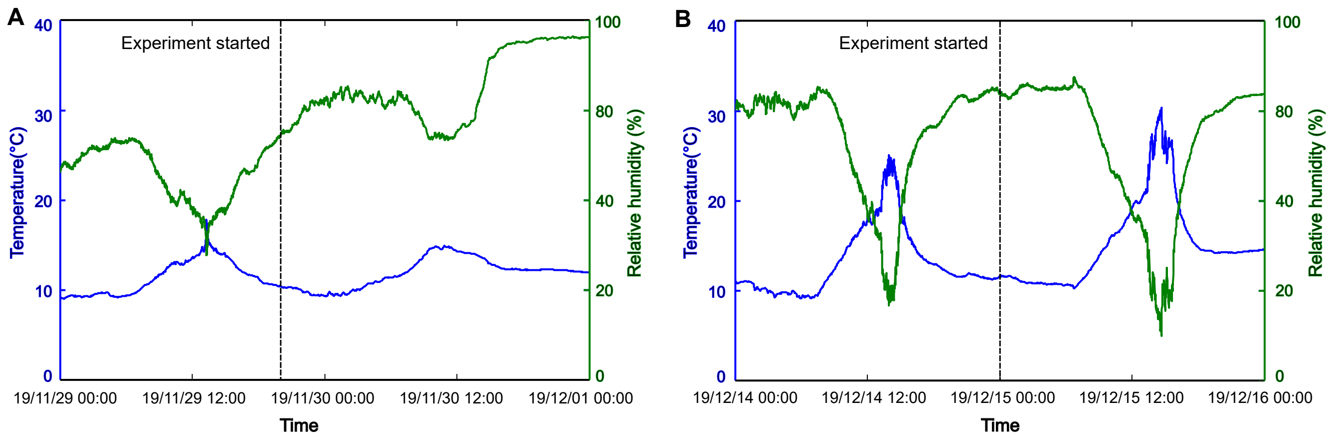

Fig. S3. Recorded environmental temperature and relative humidity. (*A*) The measurements for the hidden object of letter H. (*B*) The measurements for the hidden object of Mannequin. In experiment, we normally perform the data collections during the period of 12PM–2AM, which has stable temptation and relative humidity. This can avoid large atmospheric changes to affect the temporal and spatial fluctuations in the NLOS measurements.

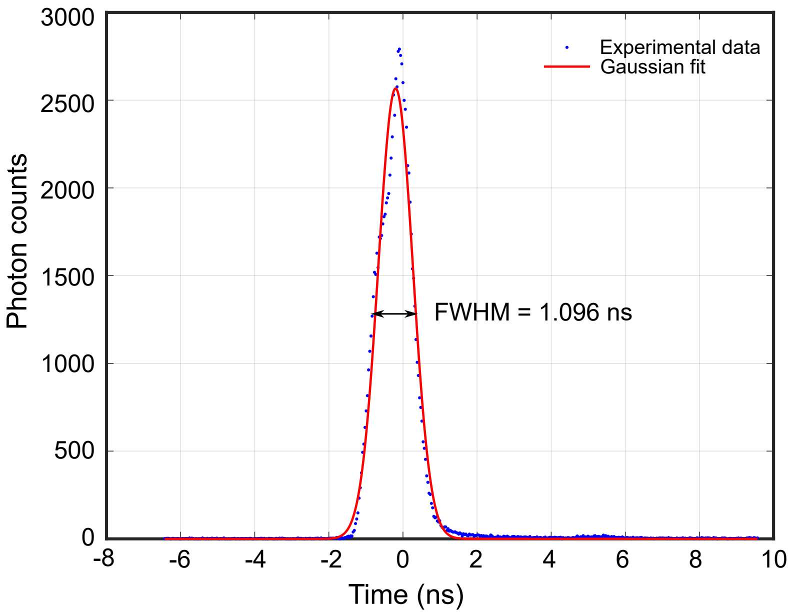

Fig. S4. NLOS imaging system's timing jitter. The time jitter of the NLOS system is measured by sending the laser pulses to the visible wall and measuring the direct line-of-sight reflections over the 1.43-km link. The experimental data is fitted by a Gaussian function. The FWHM γ_{total} is about 1096 ps, which mainly includes the temporal resolution of the InGaAs SPAD ($\sim$210 ps), the laser pulse width ($\sim$500 ps), the effective timing jitter of FOV ($2\sqrt{ln(2)}$FOV$/(\tan\psi \cdot c)$ $\sim$ 855 ps) and the estimated air turbulence and other effects. ψ = 42.3° denotes the angle between the visible wall and the transmitting light.

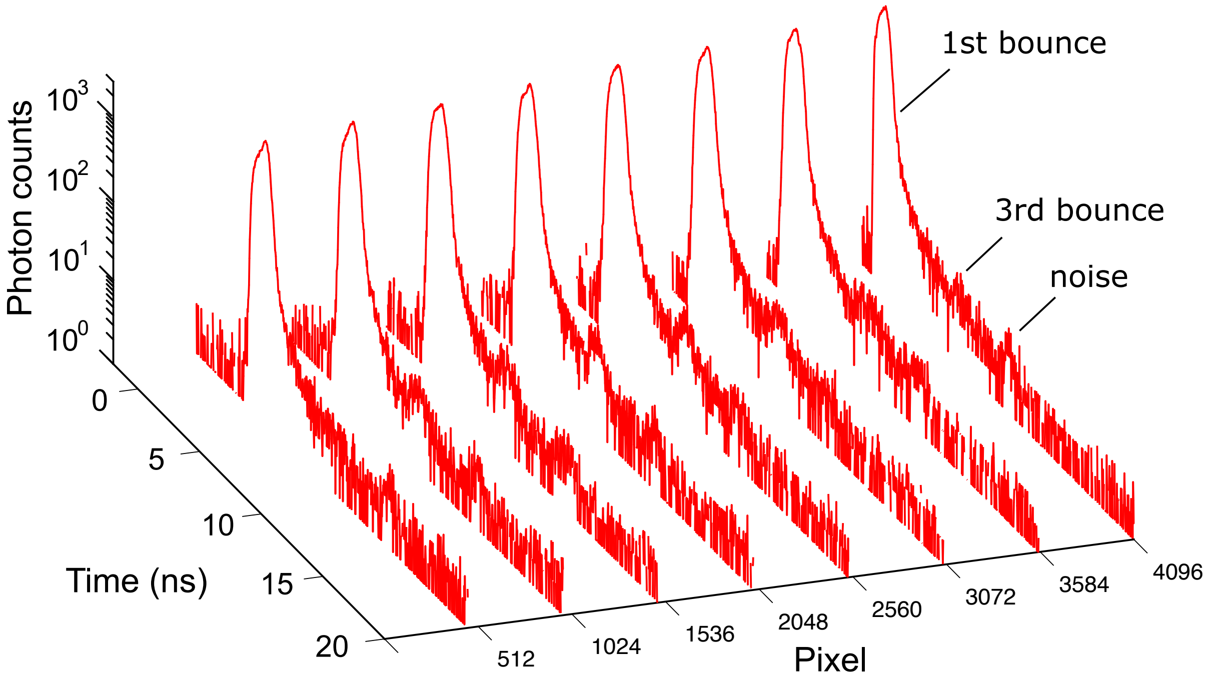

Fig. S5. A series of temporal histograms for different scanning points (pixels) observed in experiment.

 Cheng Wu, Jianjiang Liu, Xin Huang, Zheng-Ping Li, Chao Yu, Jun-Tian Ye, Jun Zhang, Qiang Zhang, Xiankang Dou, Vivek K. Goyal, Feihu Xu and Jian-Wei Pan

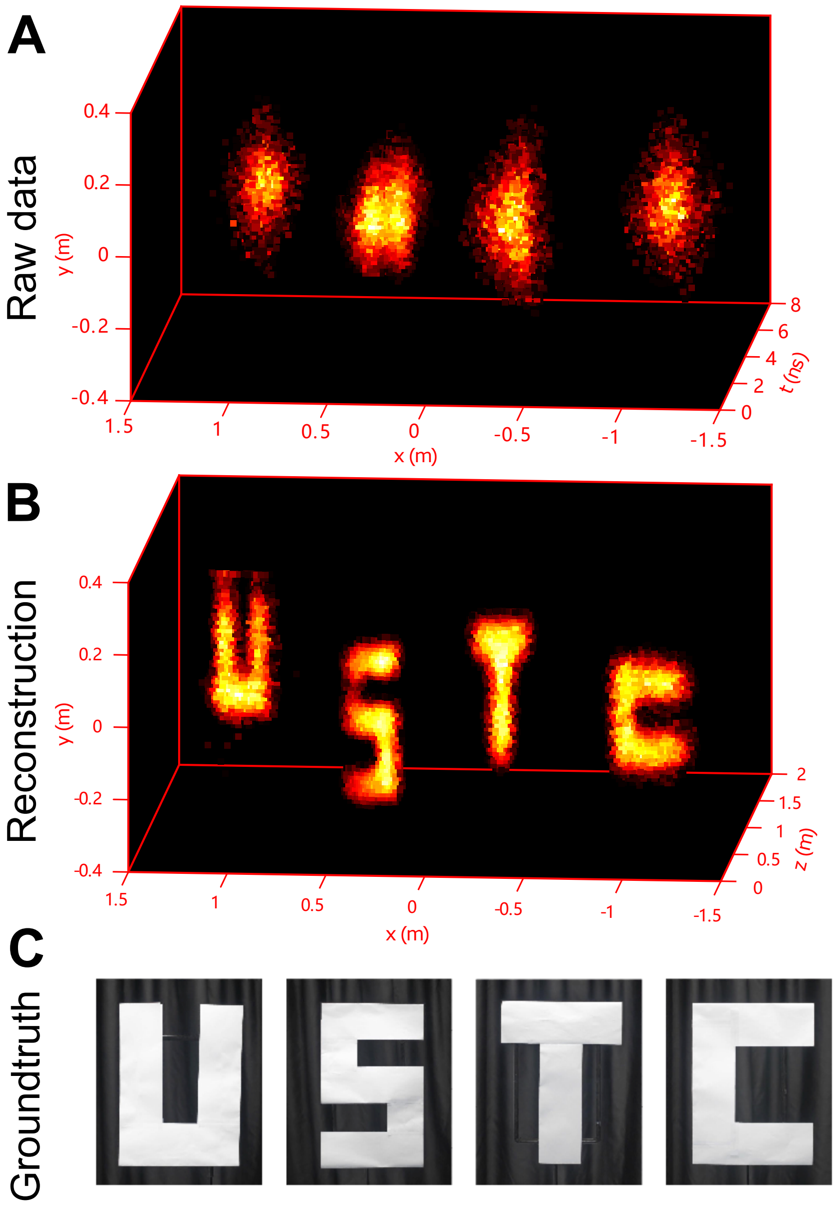

Fig. S6. Experimental NLOS imaging results for the four letters U, S, T, C, respectively.

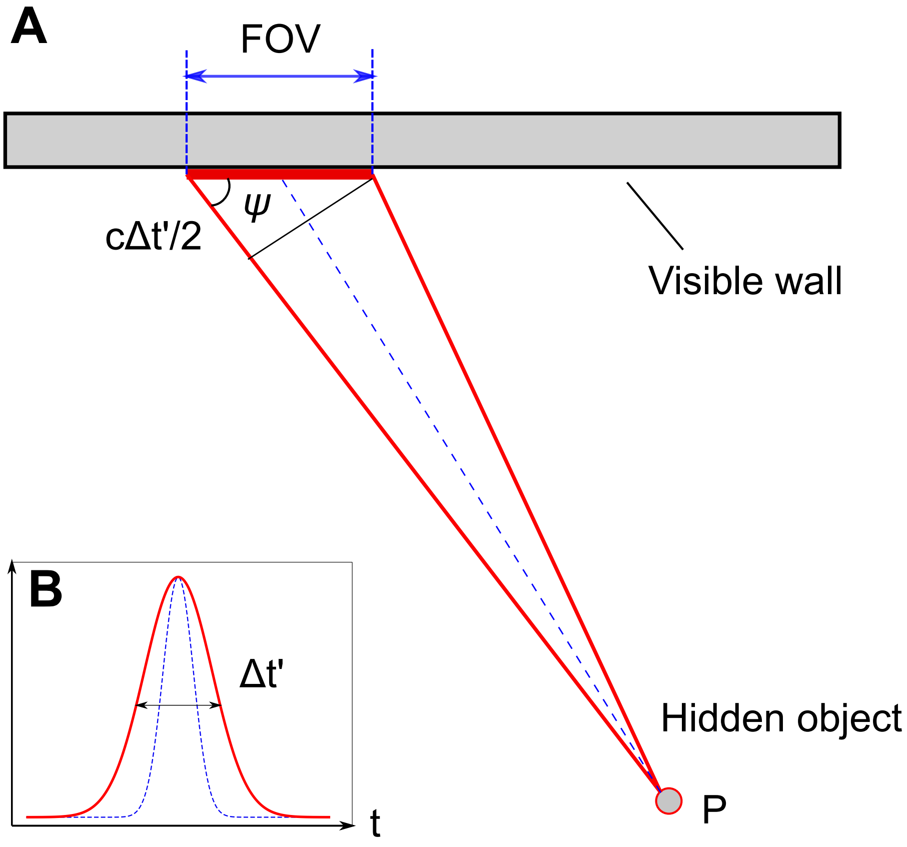

Fig. S7. Illustration of the influence of the spatial broadening to temporal response (i.e., total timing jitter). (*A*) A simplified scene geometry to explain the spatial broadening. The blue dashed line represents the ideal light transformation for the confocal system; the red solid lines demonstrate the actual light transformation when the size of the virtual source and detector can not be ignored. (*B*) Temporal response. The blue dashed line shows the response when the virtual source and detector are ideal points, and the red solid line shows the broadening response caused by the actual size of the virtual source and detector.

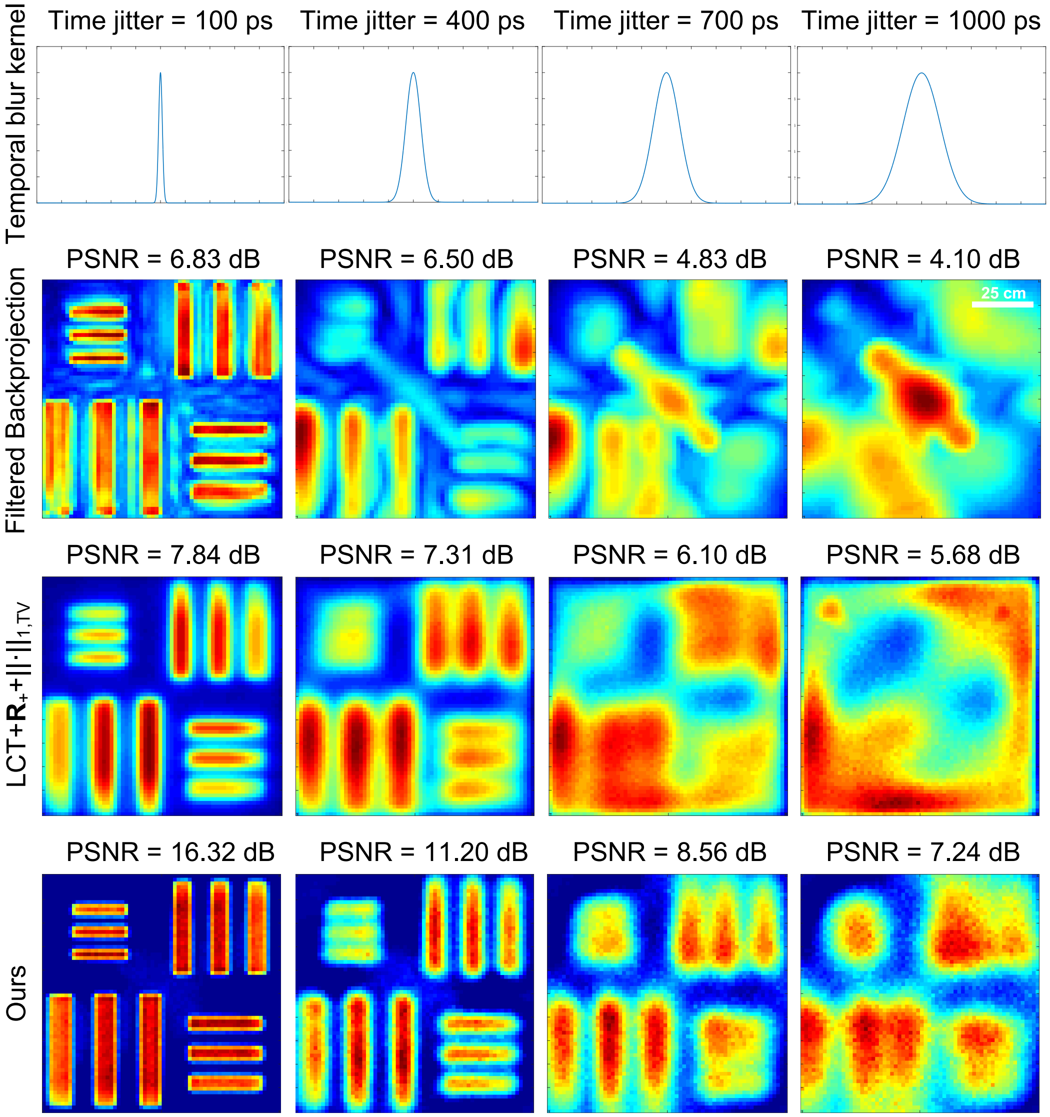

Fig. S8. Simulation results for various system timing jitters. Our approach has robust reconstructions in the case of large timing jitter.

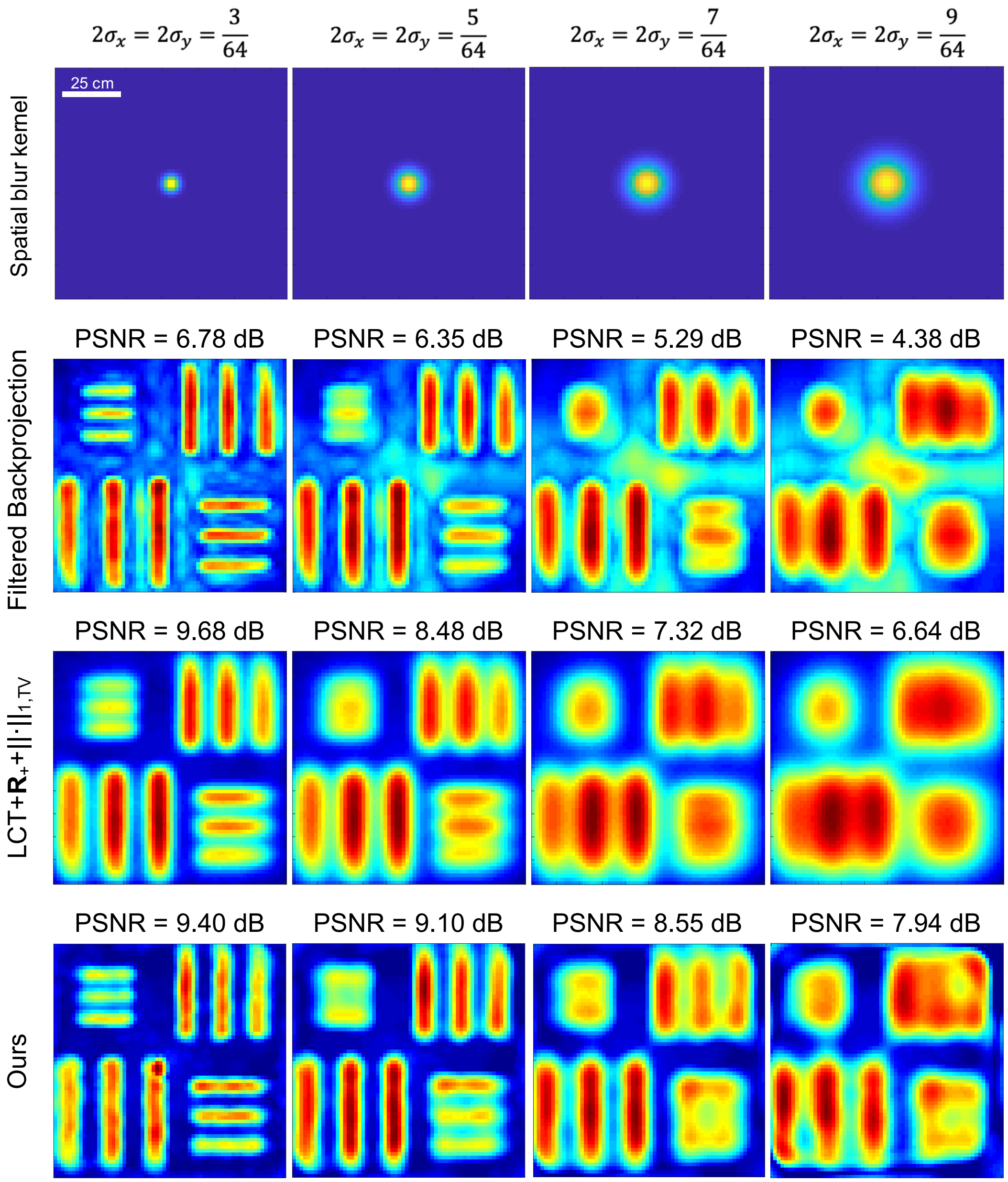

Fig. S9. Simulation results for various spatial divergences. Our approach has robust reconstructions in the case of large spatial divergences.

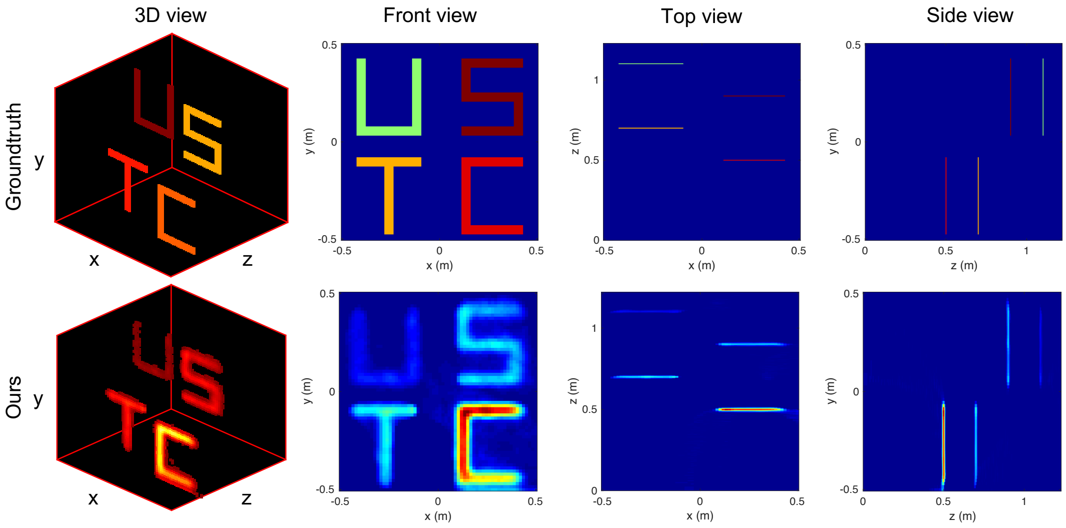

Fig. S10. Simulation results for a 3D scene of four letters.

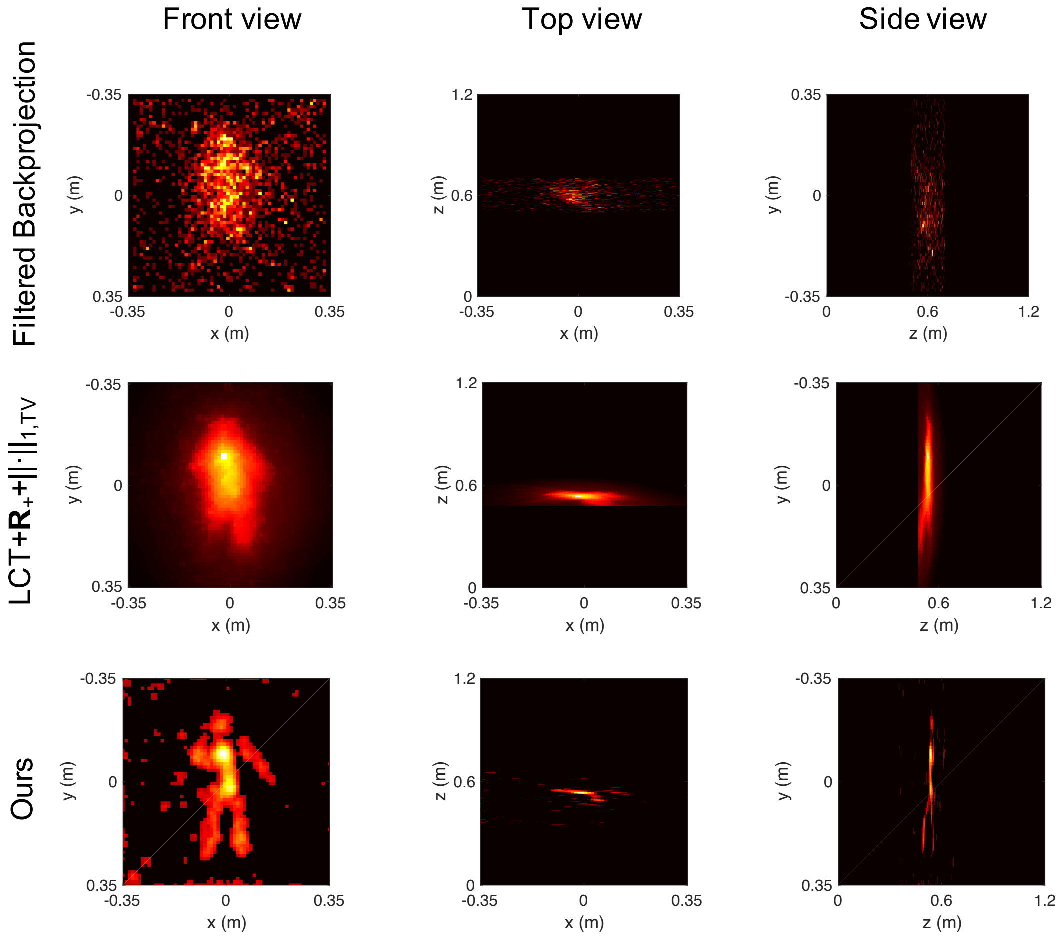

Fig. S11. Simulation results for a mannequin with public dataset in Ref. (2). We added a 400 ps time jitter and 6.6-cm FOV to simulate the spatial-temporal broadening effect.

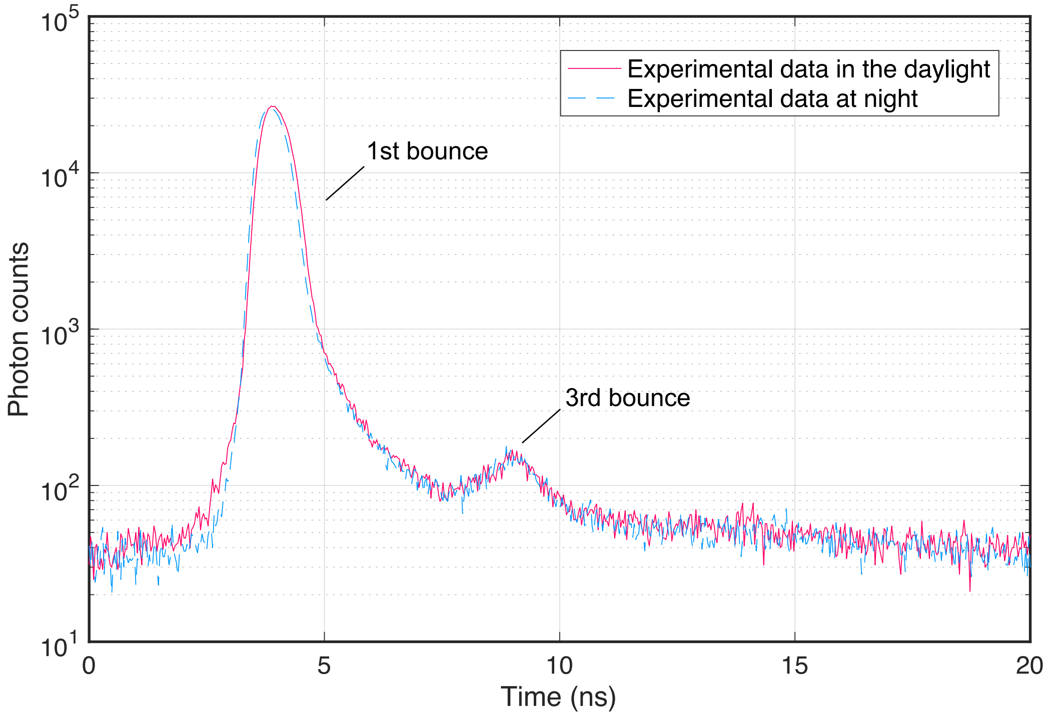

Fig. S12. Experimental measurements in daylight and night. In daylight experiment, we adopt two additional bandpass filters with FHWM = 10 nm and 1.4 nm at the center wavelength of 1550.2 nm. By using spectral filters, we observe similar SNRs for daylight and night.

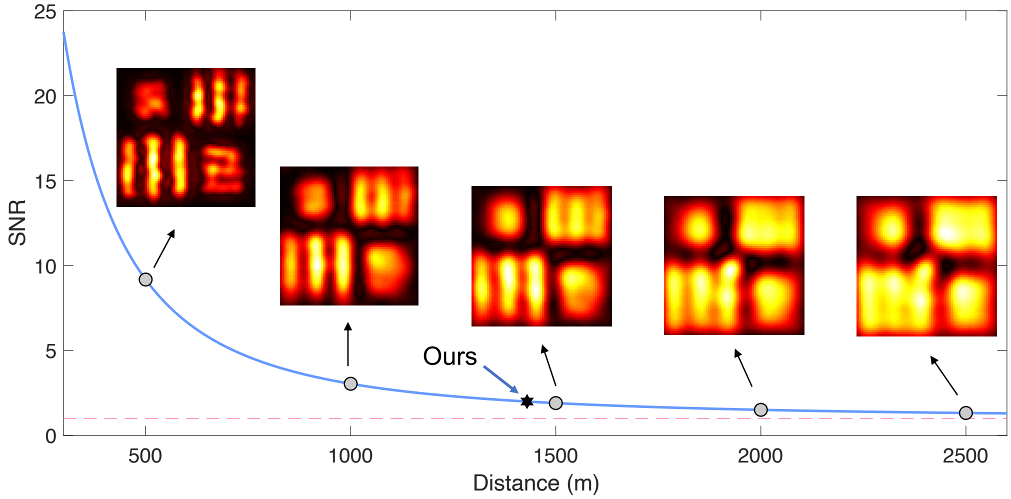

Fig. S13. Simulation results of the reconstructed chart with different standoff distances. We simulate the reconstructed results with SNR at different standoff distances, where SNR decreases quadratically with the distance. The algorithm permits a useful reconstruction with SNR as low as 1.5, and a lower SNR (or longer distance) is not sufficient for NLOS imaging. Our optical system can reach a maximal standoff distance of about 2 km to achieve a NLOS imaging resolution at 9.4 cm.

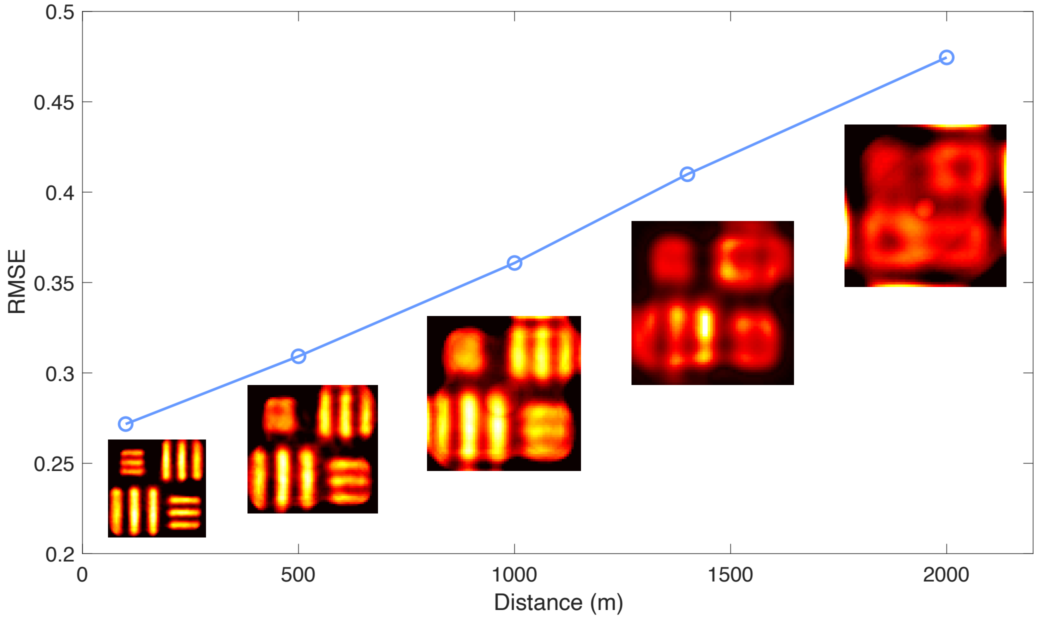

Fig. S14. Simulation results of the reconstruction accuracy with the standoff distance. With the increase of distance, the optical divergence will introduce larger spatial-temporal broadening (see Eq. (2) and 3). This will deteriorate the accuracy of the reconstructed image, where the accuracy is quantified by root-mean-square-error (RMSE).

Table S1. List of parameters for some NLOS imaging experiments. The 2nd and 3rd columns represent the first-bounce distance and the third-bounce distance. $\triangle$x and $\triangle$z represent the transverse and longitudinal resolution of the NLOS reconstruction. 'efficiency' defines as the system's detection efficiency per transmitted photon, $\frac{P_{\text{in}} l_{\text{1st}}^2 l_{\text{3rd}}^4}{P_{\text{out}}}$. 'ratio' represents efficiency ratio between ours and earlier work.

reference	l_{1st} (m)	l_{3rd} (m)	Δx (cm)	Δz (cm)	wavelength (nm)	power (P_{out}) (mW)	time jitter (ps)	efficiency	ratio
ref.(7)	0.62	0.25	0.04	~ 1	795	$N.A.$	15	$N.A.$	$N.A.$
ref.(8)	1	1.5	10	10	515	50	30	3.62×10^{-14}	2572.9
ref.(2)	2	1.15	1	1	670	0.11	60	2.08×10^{-11}	4.5
ref.(9)	2.5	0.75	2.3	2.3	532	1000	65	2.05×10^{-14}	4539.2
ref.(10)	1	1	~ 3	2.2	532	1000	70	4.30×10^{-14}	2169.2
This work	1430	0.75	~ 9.4	~ 9.4	1550	300	1096	9.32×10^{-11}	1

Movie S1. A supporting video illustration of the experiment.

References

1. W Wagner, A Ullrich, V Ducic, T Melzer, N Studnicka, Gaussian decomposition and calibration of a novel small-footprint full-waveform digitising airborne laser scanner. *ISPRS J. Photogramm. Remote. Sens.* **60**, 100–112 (2006).
2. M O'Toole, DB Lindell, G Wetzstein, Confocal non-line-of-sight imaging based on the light-cone transform. *Nature* **555**, 338 (2018).
3. AM Pawlikowska, A Halimi, RA Lamb, GS Buller, Single-photon three-dimensional imaging at up to 10 kilometers range. *Opt. express* **25**, 11919–11931 (2017).
4. ZP Li, et al., Super-resolution single-photon imaging at 8.2 kilometers. *Opt. Express* **28**, 4076–4087 (2020).
5. RJ Thomas, et al., A procedure for multiple-pulse maximum permissible exposure determination under the z136.1-2000 american national standard for safe use of lasers. *J. Laser Appl.* **13**, 134–140 (2001).
6. ANS Institute, *American national standard for safe use of lasers.* (Laser Institute of America), (2007).
7. A Velten, et al., Recovering three-dimensional shape around a corner using ultrafast time-of-flight imaging. *Nat. Commun.* **3**, 745 (2012).
8. M Buttafava, J Zeman, A Tosi, K Eliceiri, A Velten, Non-line-of-sight imaging using a time-gated single photon avalanche diode. *Opt. Express* **23**, 20997–21011 (2015).
9. X Liu, et al., Non-line-of-sight imaging using phasor-field virtual wave optics. *Nature* **572**, 620–623 (2019).
10. DB Lindell, G Wetzstein, M O'Toole, Wave-based non-line-of-sight imaging using fast f–k migration. *ACM Transactions on Graph.* **38** (2019).
